# Supplementary material for: Comprehensive Annotation and Functional Exploration of MicroRNAs in Lettuce
Source: Front Plant Sci. 2021 Dec 24;12:781836. doi: 10.3389/fpls.2021.781836 (PMC8739914; doi:10.3389/fpls.2021.781836)
Supplement: Supplementary file 2 [file Data_Sheet_8.ZIP › GSM2230749.plot/Lsa-miRN48_Lsat_1_v5_gn_9_41220.1_2776_TPlot.pdf]

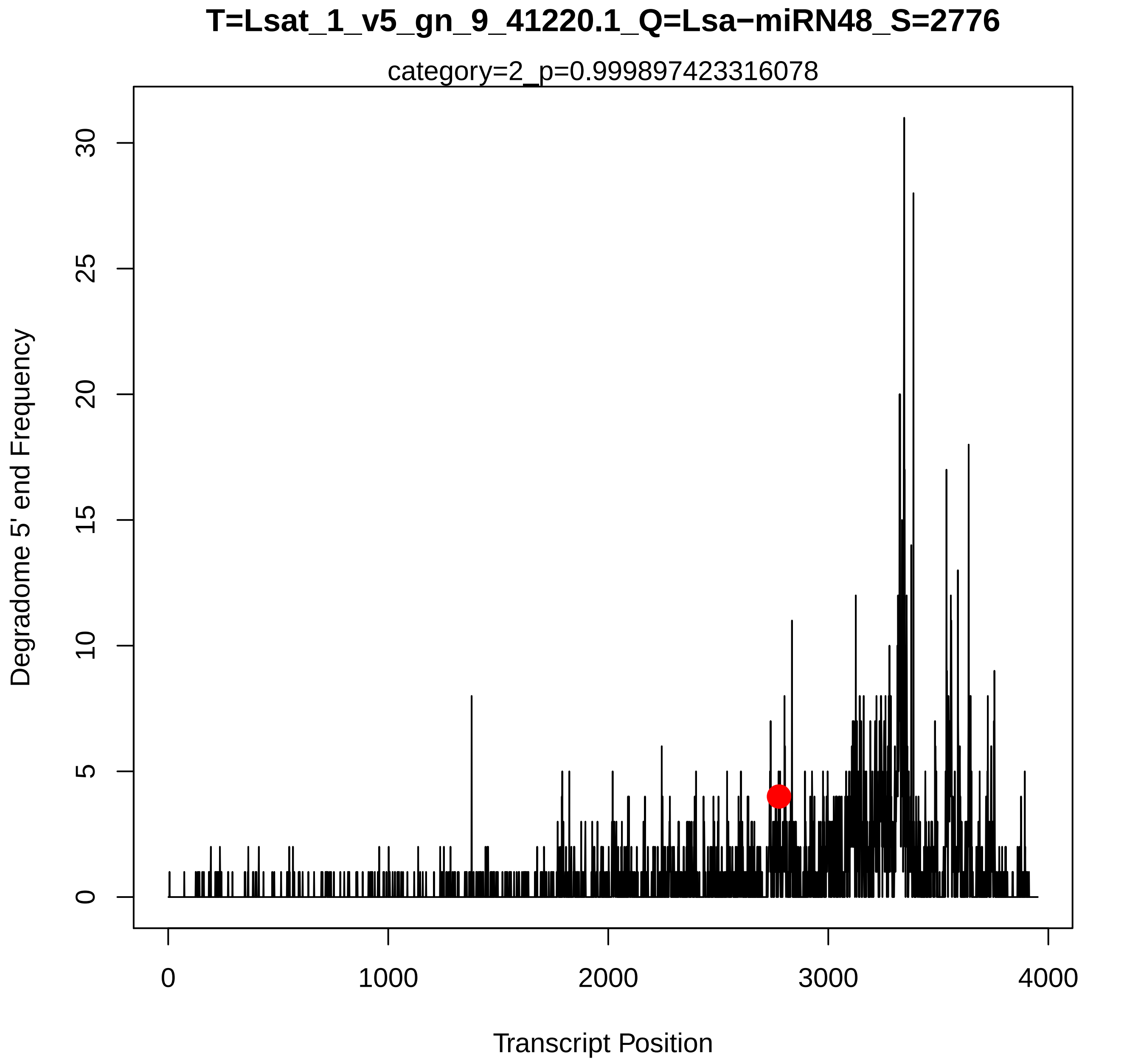
T=Lsat_1_v5_gn_9_41220.1_Q=Lsa−miRN48_S=2776
category=2_p=0.999897423316078
Degradome 5' end Frequency
0
5
10
15
20
25
30
Transcript Position
0
1000
2000
3000
4000
